# Botanical identification of medicinal roots collected and traded in Morocco and comparison to the existing literature

**DOI:** 10.1186/1746-4269-9-59

**Published:** 2013-08-15

**Authors:** Abderrahim Ouarghidi, Gary J Martin, Bronwen Powell, Gabrielle Esser, Abdelaziz Abbad

**Affiliations:** 1 University of Cadi Ayyad, Marrakech, Morocco; 2 Global Diversity Foundation, Marrakech, Morocco; 3 Centre for International Forestry Research, Bogor, Indonesia; 4 University of British Columbia, Vancouver, British Columbia, Canada

**Keywords:** Medicinal plant, Root, Voucher, Herbalist, Collector, Ethnobotany, Marrakech, Plantes médicinales, Racines, Voucher, Herboriste, Collecteur, Ethnobotanique, Marrakech

## Abstract

**Background:**

A literature review revealed heavy reliance on a few key publications for identification of medicinal plant species from local or vernacular names and a lack of citation of voucher specimens in many publications. There is a need for more reliable and standardized data on the identity of species used for medicine, especially because local names vary from region to region. This is especially true in the case of medicinal roots, for which identification of species is difficult. This paper contributes to existing data on the species sold as medicinal roots (and other underground plant parts such as bulbs, corms, rhizomes and tubers) in Morocco.

**Methods:**

Data were collected in collaboration with herbalists in Marrakech and collectors in rural regions near Marrakech where species are collected from the wild. The ethno-medicinal uses of these species were also recorded.

**Results:**

We identified the vernacular names for 67 medicinal roots (by free listing) used to treat a variety of human diseases. We were able to collect and identify one or more species for 39 of the recorded vernacular names. The ones we were not able to identify were either imported or no longer available in the markets. We collected more than one species for some of the vernacular names for a total of 43 species. We identified six new vernacular names and four species which had not been previously described in the literature. Our botanical identification matched at least one of the names listed in the literature 63% of the time and did not match any species listed in the literature 37% of the time. Of the three most commonly cited pieces of literature we compared to, we found the greatest overlap with the broader, more comprehensive work of Bellakhdar 1997 (as opposed to Benchâabane and Abbad 1997 which worked in a similarly focused geographical area). However there was only 63% agreement between Bellakhdar 1997 and our botanical identifications, and 29% of the time our identification didn’t match even the genus of any of the species listed in any of the 3 most commonly cited pieces of literature.

**Conclusions:**

More rigorous methodology and reporting are needed for medicinal plant research in Morocco. This will ensure that studies are comparable, help to protect traditional medicine users from negative health effects, and, support efforts to conserve overharvested wild medicinal plants.

## Abstract (fr)

**Résumé**

Une revue de la littérature a révélé que l’identification des plantes médicinales (à partir des noms vernaculaires locaux) reste dépendante d’un nombre limités de publications. Une standardisation et une mise en place d’une base de données plus fiable sur l’identité des plantes médicinales utilisées en médecine deviennent un besoin incessant; d'autant plus que les noms locaux peuvent varier d'une région à l'autre. Cela est particulièrement vrai pour les espèces dont les parties souterraines sont utilisées. Cette étude vise à contribuer aux données existantes sur les espèces commercialisées sous forme de racines médicinales (et autres organes souterrains des plantes) dans les marchés de Marrakech.

**Méthodes**

Les données ont été collectées en collaboration avec les herboristes et les collecteurs à Marrakech et dans plusieurs de ses régions limitrophes où les espèces ont été collectées dans leur milieu naturel. L’utilisation ethno-médicinale locale pour chaque espèce médicinale a été enregistrée.

**Résultats**

Nous avons inventorié (par la liste libre) les noms vernaculaires correspondant à 67 racines médicinales utilisées pour traiter une panoplie de maladies humaines. Une ou plusieurs espèces végétales ont été collectées et identifiées correspondant à 39 des noms vernaculaires des racines inventoriés. Les espèces que nous n'étions pas en mesure d'identifier étaient essentiellement soit importées ou ne sont plus disponibles sur le marché. Six noms vernaculaires et quatre espèces végétales, qui n’avaient jamais été précédemment décrits dans la littérature, ont été identifiés pour la première fois dans ce travail. L’identification botanique rapportée dans ce travail correspondait à 63% des noms des espèces énumérées dans la littérature et inversement diffère de 37% de ceux précédemment décrits. L’analyse comparative avec les trois littératures les plus fréquemment citées, a montré une grande concordance avec Bellakhdar (1997), considéré comme le travail le plus complet (par opposition à Benchaâbane et Abbad (1997) qui ont travaillé dans la même zone géographique). Toutefois, cette concordance entre notre identification botanique et celle de Bellakhdar (1997) n’était que de 63%, alors que 29% des cas nous n’avons pas trouvé une correspondance même au niveau des genres des espèces décrites par les trois littératures les plus fréquemment citées.

Plus de méthodologie rigoureuse et de publications sont nécessaires pour la recherche en plantes médicinales au Maroc. Il parait évident que l’identification botanique des échantillons provenant de différentes régions du pays soutiendra le développement d'une base de données plus fonctionnelle des plantes médicinales et de leurs noms vernaculaires locaux.

## Background

During the last decade, medicinal plants and their products have attracted world-wide interest due to the growing recognition of natural products and the potential for drug discovery [1]. Many populations rely on medicinal plants because they are easily available at an affordable price. Morocco has high cultural diversity, a rich traditional medical system and associated traditional knowledge, and high rates of biodiversity which provide a diversity of medicinal plants. It has been estimated that approximately 7000 plant species and sub-species grow wild in Morocco, 950 of which are endemic [2,3]. Among these, many species are aromatic or medicinal plants and are used locally in Morocco’s rich and widely used traditional medicine system. It has been estimated that about 231 local plant species present phytotherapeutic properties used by the local population to treat a variety of diseases [4,5].

Subterranean or underground organs of medicinal plants locally referred to as *laaroug* which means “roots” in Moroccan Arabic, play a central role in the Moroccan pharmacopoeia. One third of plant materials used in Moroccan traditional medicine is derived from underground organs [6] such as roots, bulbs, tubercles, and rhizomes (henceforth referred to as roots). Most of these are sold in herbal market stalls in a dried state which makes identification very difficult. Previous research has shown that both lay people and experts have more difficulty identifying medicinal roots than most other plant parts [7] and that not all herbalists have the necessary skill to identify medicinal root species accurately.

There is a large and growing body of research on medicinal plants and their pharmacological properties in Morocco. However, much of this research is hindered by reliance on previous publications to identify species from a given local (or vernacular) name. This problem is exacerbated by the fact that many medicinal plants species have multiple local names to describe them, and, inversely, local names can refer to multiple species [1,8]. In order to support future research and enhance research accuracy, it is necessary to establish a better and more reliable knowledge base of the identity of medicinal species, especially those used for their roots which are particularly prone to confusion. The present paper presents a brief literature review highlighting the need for additional primary botanical identification of medicinal plants in Morocco followed by the botanical identity and ethno-medicinal uses of species used for their roots, collected from the wild in southern Morocco and sold in the markets of Marrakech.

## Methods

### Study area

**Figure 1:**
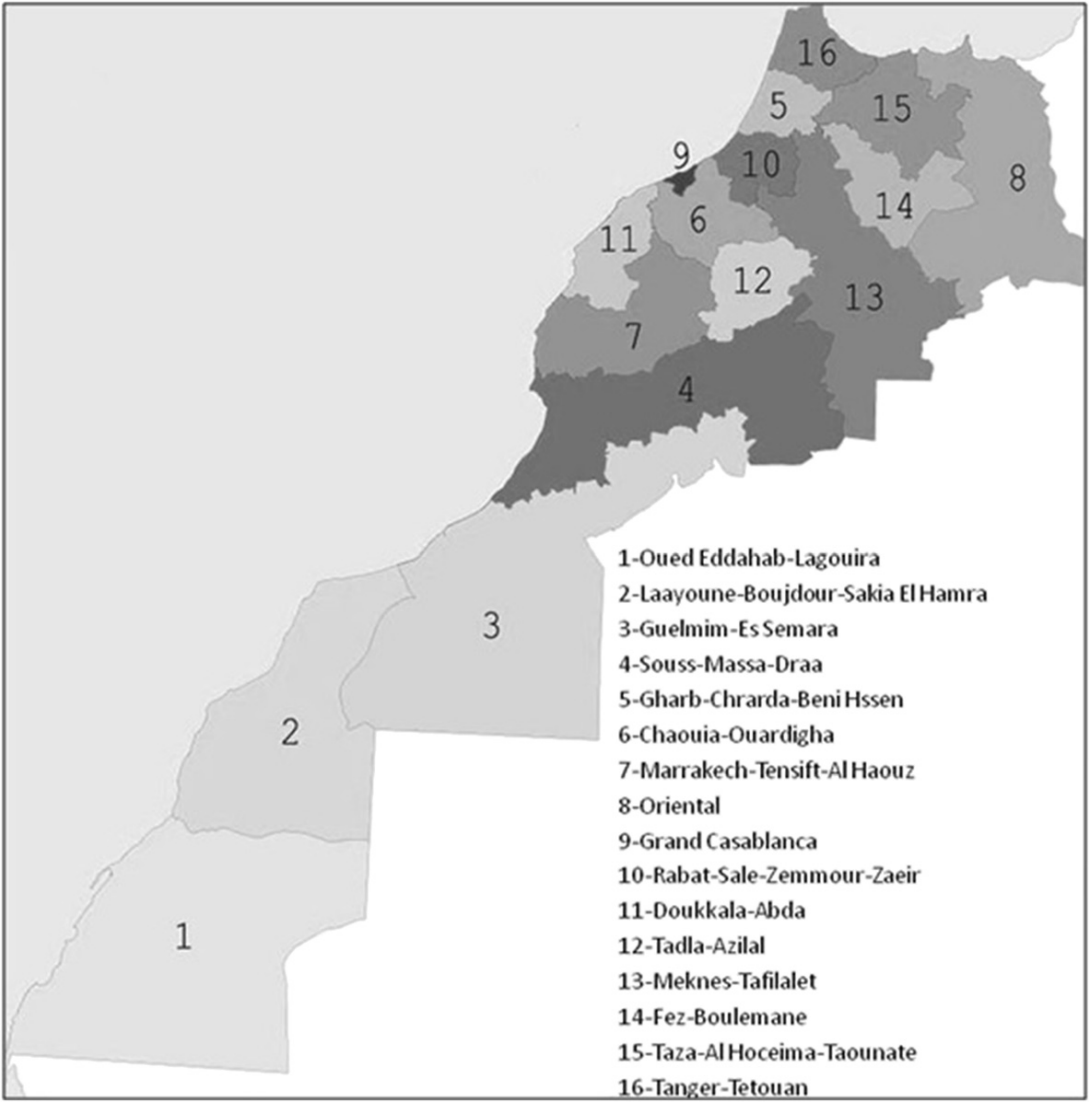
**Map of Morocco with provinces numbered for reporting location of literature review studies (see Table**1**).**

**Figure 2:**
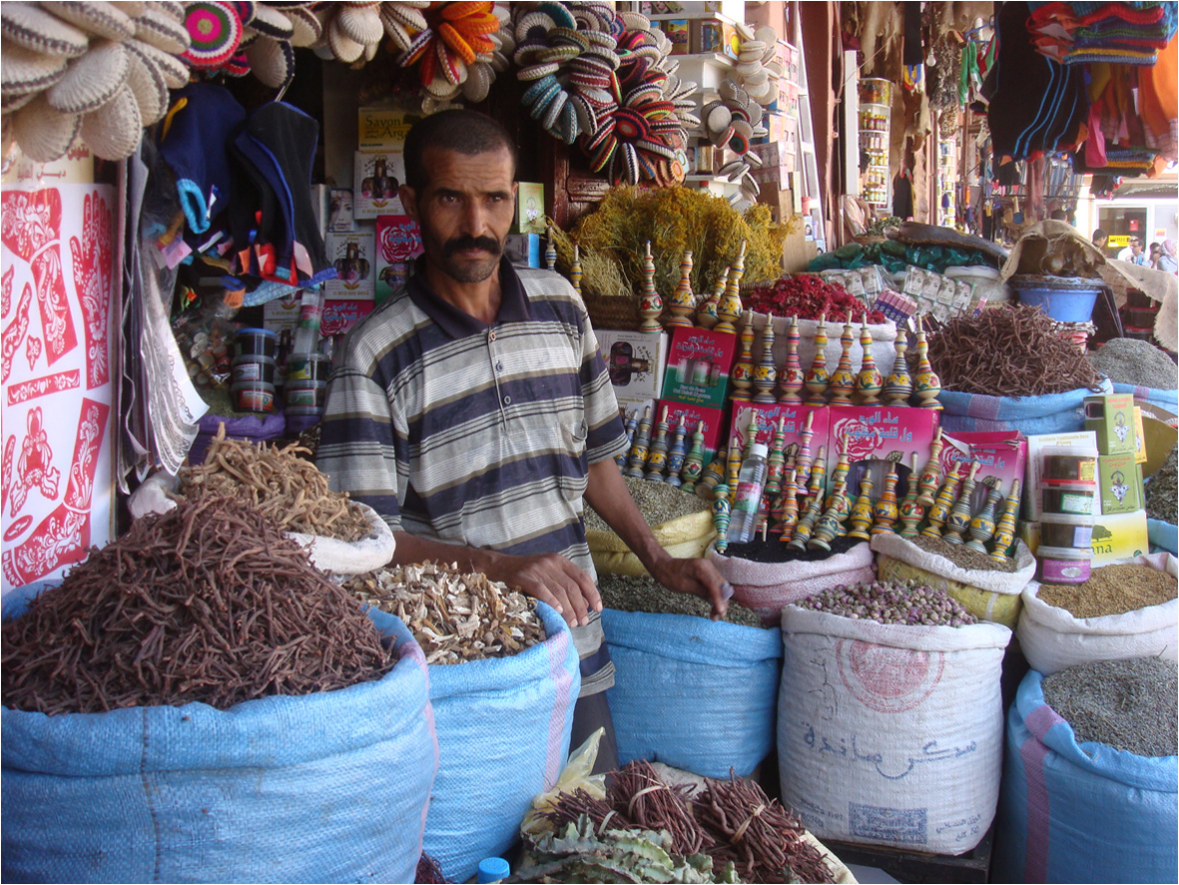
Bags containing single medicinal roots and herbal mixtures in the Marrakech herbal market (Photo: A. Ouarghidi).

**Figure 3:**
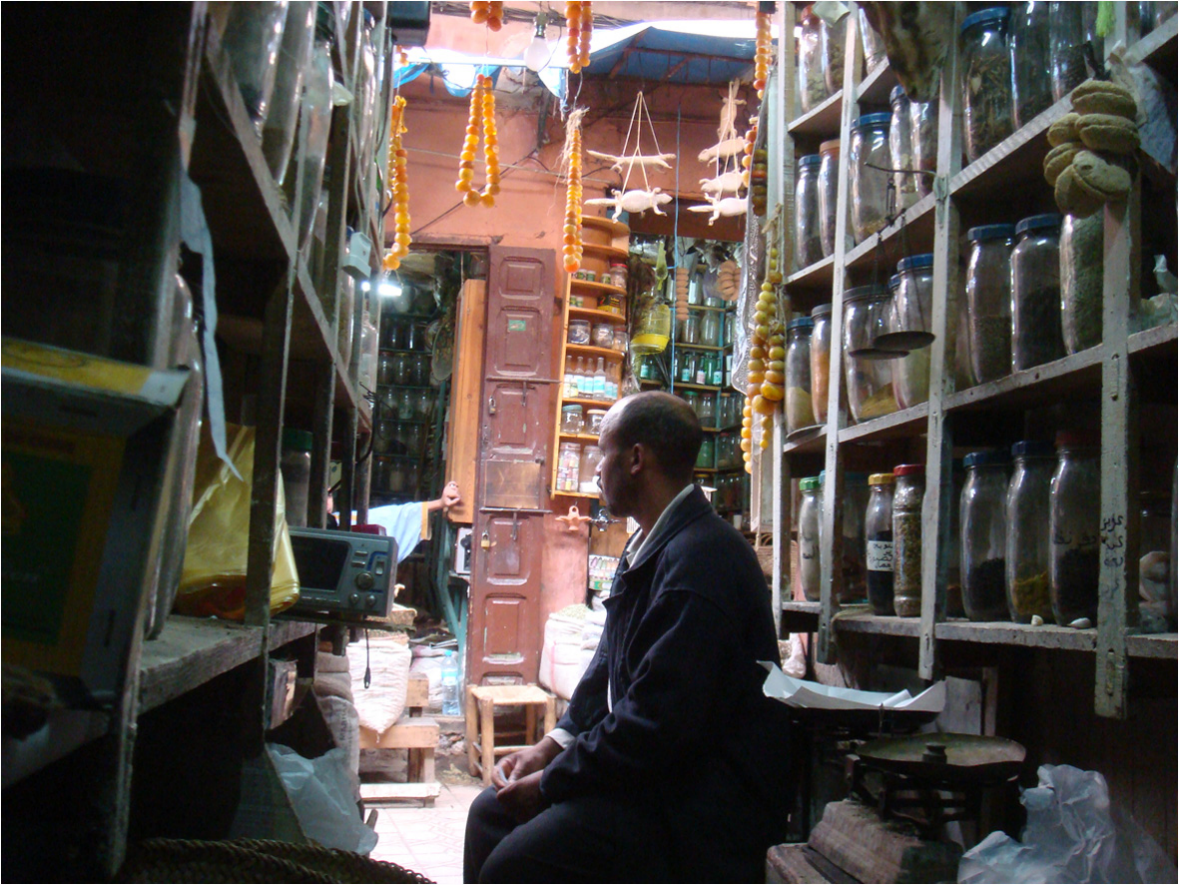
Herbalist’s store in the Marrakech herbal market (Photo: A. Ouarghidi).

This paper includes a literature review from research conducted across Morocco (Figure 1). The roots identified in our botanical work were all reported to be sold in the herbal markets of Marrakech by herbalists and collected in surrounding rural regions (Figures 2 and 3). The seven rural collection sites, in the surrounding plains and the High Atlas Mountains, were between 10 and 240 km from Marrakech (Larbaa Tighdouine, Oukaimden, Touama, Tadart, Ait M’hamed, Sebt Aguerferouane, Ben Guerir, Asni, Tensift, and were in Marrakech and Azilal provinces (7 and 12 on the map in Figure 1). The collection site ecosystems were arid to semi-arid, similar to others found around the Mediterranean basin. These ecosystems are characterized by a rich endemic flora due, in part, to geographical variation providing a variety of bio-climates and habitat heterogeneity. The seasonal harvest of wild medicinal plants provides an important source of income for participating families in rural areas. These families play an important role in the conservation and management of these resources.

### Literature review

A systematic literature review was conducted on Google Scholar and Pub Med using search terms “Morocco”, “Maroc”, “North Africa”, “Medicinal Plant”, “Herbal”, “Traditional Medicine” or “Ethnobotany” for papers in French or English, with no restriction on date of publication (January 2012). A hundred and thirty six potential papers were identified before applying exclusion criteria to narrow the list. Following systematic review guidelines the inclusion / exclusion criteria were set in advance by 2 researchers with the aim of including only papers that would realistically be expected to include botanical identification and vouchers. Exclusion criteria included: papers on only one species (and papers with species identified in the title), papers which looked only at pharmacological actions and papers on cultivated or food species. After excluding papers with the above criteria, 26 papers were identified, obtained, and reviewed. We looked at how researchers had identified medicinal plants: the use of botanical keys, name of the botanists who did the identification and the affiliation of the botanist or the authors involved in identification.

### Botanical identification of local names

Field work was undertaken from 2007–2010 to gather data on species where the root is used in traditional medicine in the markets of Marrakech. Verbal consent was obtained from all herbalists and collectors after research purposes had been explained in detail. We selected 15 herbalists who were the most knowledgeable about medicinal roots, based on our previous extensive work. These 15 herbalists were asked to list all the medicinal roots traded in southern Morocco. Together they identified 67 medicinal roots (and subterranean plant parts) by their local or vernacular names. These roots were then traced through the market chain to various collection sites in rural areas surrounding Marrakech. Twenty three collectors were identified and voucher specimens were collected with them during their normal collecting activities. Specimens matching local names were collected in each of the sites where collection was taking place (one or more specimens for each vernacular name). Botanical identification of plants was done by Abderrahim Ouarghidi, under the supervision of Dr Mohamed Ibn Tattou and Dr Mohamed Fennane from the Scientific Institute of Rabat (ISR). Collected vouchers were compared with specimens in the Herbarium of ISR. Flore de L’Afrique du Nord [9] and Petite Flore des Régions arides du Maroc occidental [10] were the botanical keys consulted during identification. Voucher specimens were deposited in the Natural History Museum of Marrakech.

### Comparison to species identified in the literature

The botanical species identified for each local name were compared with those previously reported in the most commonly cited literature. The literature review identified Bellakhdar “La Pharmacopée Marociane Traditionelle: Médicine Arabe Ancienne et Savoirs Populaires” [11], Benchâabane and Abbad “Les plantes medicinales commercialisées à Marrakech” [12], and Boulos “Medicinal Plants of North Africa” [13] as the most commonly cited sources for botanical identification of vernacular names in medicinal plant research papers from Morocco (on Google Scholar, Boulos [13] appears as cited 319 times, Bellakhdar [11] 251 times, January 2012). We compared the species we collected to species cited for the same vernacular name by these three ‘expert texts’. Because these texts do not cite voucher specimens we were not able to differentiate between incorrect identification and cases where vernacular names refer to multiple botanical species (a likely scenario as these texts cover a wide geographic region).

### Traditional Use information and availability

Information was collected using structured and semi-structured interviews with over 80 herbalists in Marrakech to identify the traditional uses for each medicinal root and its relative abundance (availability) in the market. Roots were classified based on availability: rare, common and abundant.

## Results

### Literature review of medicinal plant identification in Morocco

Of the 26 papers we reviewed, 42% cited one or more botanical key used for the identification of collected species, 42% reported the name of the botanist(s) carrying out the identification, and 61.5% the botanist(s)’ affiliation (Table 1). Although 58% papers reported the collection of voucher specimens, only 35% reported voucher numbers. The remaining papers used existing literature to identify species from vernacular names, rather than using a scientific identification process. Of the provinces of Morocco (listed in Figure 1), Chaouia-Ouardigha, Grand Casablanca, Doukkala-Abda (with only one study in each), the three Saharaian provinces and Azilal (with only 2 studies in each), and Marrakech, Oriental and Fez-Boulemane (with only 3 studies in each) were the least studied provinces. Meknes-Tafilalet was the most studied province (with 30% of the studies we reviewed having been conducted there), followed by Rabat, Gharb, Taza-Taounate and Tanger (with 4, 5 or 6 studies in each).

**Table 1:** Summary of 26 papers on medicinal plants in Morocco including methods used for botanical identification (use of a botanical key, name of the botanist, institution of the botanist or authors and number of species identified)

**Paper**	**Methodology**	**Keys**	**Botanists**	**Institutions**	**Voucher mentioned**	**Voucher N° reported**	**Number of Plant Species Identified**	**Study area**
Bellakhdar et al. [4]	Interviews and specimen collection with traditional healers	Yes	Yes	Yes	Yes	Yes	231	1, 2, 3, 4, 5, 6, 7, 8, 9, 10, 11, 12, 13, 14, 15, 16
Benkhnigue et al. [14]	Ethnobotanical investigations, based on 280 interviews, conducted during two periods in 2006 and 2007.	Yes	No	No	No	No	149	5
Eddouks et al. [15]	Ethnobotanical information was obtained from 280 local residents.	No	Yes	Yes	Yes	Yes	92	13
El Amrani et al. [16]	Ethnobotanical study carried out with herbalists, traditional healers and patients. Samples were purchased from herbalists stores.	No	No	No	No	No	42	13
El Mansouri et al. [17]	Samples of plants used locally were harvested on land and/or requested from herbalists.	Yes	No	No	No	No	109	13
El Rhaffari [18]	Ethnobotanical study carried out with herbalists, traditional healers and patients (leishmaniose cutanée)	Yes	No	No	Yes	No	61	13
El Rhaffari and Zaid, [19]	Surveys carried out with herbalists, traditional healers and consumers.	No	Yes	Yes	Yes	No	215	13
El-Hilaly et al. [20]	Standard Ethnobotanical survey of the Taounate with (1) those who knew and/or used plants for medicinal purposes and (2) those who used plants and plant products for commercial purposes (plant collectors, wholesalers, retailers).	No	Yes	Yes	Yes	No	102	15
Ennabili et al. [21]	295 interviews at 29 sites using a structured questionnaire form.	Yes	Yes	Yes	Yes	No	78	16
González-Tejero et al. [22]	In Ouezanne region, 87 Semi-structured interviews and participant observation of 72 informants.	Yes	Yes	Yes	Yes	No	100	5
Hseini et al. [23]	An inventory of medicinal plants was carried out in the region of Rabat with traditional healers.	Yes	Yes	Yes	No	No	280	10
Jouad et al. [24]	Interview of 25 traditional herbal healers and a total of more than 1153 patients who use medicinal plants for treatment.	Yes	Yes	Yes	Yes	Yes	90	14
Kabbaj et al. [25]	Information on the anti-cancer plants used, method of preparation, dosage, treatment duration and observance during phytotherapy.	No	No	No	No	No	55	10
Khabbach [26]	1) Survey of 291 of the local population interviewees, 2) Field verification surveying	Yes	Yes	Yes	No	No	140	15
Lahsissene and Kahouadji [27]	Two ethnobotanical surveys were carried out with the local community of the Zaër area (2002–2003 et 2003–2004).	No	No	No	No	Yes	228	10
Lahsissene et al., [28]	Ethnobotanical study carried out with traditional healers, herbalists and users of medicinal plants.	Yes	No	Yes	No	No	228	10
Larhsini et al. [29]	12 plant species selected for testing based on folk-medicine reports	No	No	No	No	No	12	Not reported
Larhsini et al. [30]	Plants were collected in the south of Morocco. Antipyretic activity of three medicinal species.	No	No	Yes	Yes	Yes	3	4
Markouk et al. [31]	Plant materials were authenticated by a single academic expert and were then tested for insect repellent abilities by separating specific substrates such as leaves or aqueous extracts	No	No	Yes	Yes	Yes	4	4, 10, 12
Mehdioui and Kahouadji [32]	Interviews were carried out with local community of Imi n’Tlit.	No	No	No	No	No	42	7
Merzouki et al. [33]	Each plant species was identified by Ethnobotanical survey/collection of plants local herbalists and uses were indexed interviews with 785 persons	No	No	Yes	Yes	Yes	186	16
Mouhajir et al. [34]	The plants were collected in several regions of Morocco in the summers (Atlas & Rif Mountains and Sahara) of1997–2000.	No	No	Yes	Yes	No	75	1, 2, 3, 4, 5, 7, 8, 13, 14, 15
Salhi et al. [35]	Ethnobotanical survey carried out with the local community.	No	No	No	No	No	55	5
Sqalli et al. [36]	31 species were collected for assessing their Antimycobacterien effect.	No	No	No	Yes	No	31	16
Tahraoui et al. [37]	Ethno-pharmacological survey given to 400 who knew about and/or had used medicinal plants, including suppliers and vendors	Yes	Yes	Yes	Yes	Yes	64	13
Ziyyat et al. [38]	Interviews were carried out with people suffering, both diabetes and high blood pressure	No	Yes	Yes	Yes	Yes	42	8, 15

### Identification of medicinal roots

We identified 67 medicinal roots (by free listing of local name) used to treat a variety of human diseases. Nine vernacular names appeared on the free lists of at least 10 of the 15 herbalists interviewed. More than 35% of vernacular names appeared on 2 or fewer of the herbalists’ free lists. Medicinal uses listed were diverse, including: rheumatism, cold, gaining weight, aphrodisiac, female reproductive ailments, stomach and intestine problems, skin diseases, and hair problems (Table 2).

**Table 2:** List of medicinal roots and their ethno-medicinal uses in Marrakech region (in order of frequency on 15 herbalists’ free lists)

**Vernacular names**	**Botanical identification**	**Family**	**Voucher**	**Frequency**	**Uses**	**Reported availability**	**Other scientific names listed in the literature**
Foua	*Rubia peregrina* L.	Rubiaceae	795A	12	Hepatitis, Liver problems, Tonic, Gain weight	Common	*Rubia peregrina* L.^a,b,c^
							*Rubia tinctorum* L.^a,b,c^
							*Galium odoratum* (L.) Scop.^b^
Serghina (Tasserghint)	*Petrorhagia illyrica* (Ard.) P.W. Ball & Heywood, *Corrigiola telephiifolia* Pourret	Caryophyllaceae	866A, 867A	12	Gain weight, Appetizer, Incense, Headache, Migraine	Common	*Corrigiola telephiifolia* Pourret ^a,b,c^
Kherchouf (Kherchouf Beldi)	*Cynara cardunculus* L.	Asteraceae	470K	11	Abdominal pain	Common	*Cynara cardunculus* L. ^a,b,c^
							*Cynara humilis* L.^b^
							*Cynara scolymus* L.^b^
L’guseb	*Phragmites communis* Trin.	Poaceae	836A	11	Hair problems	Common	*Phragmites communis* Trin*.*^a^*Phragmites australis* (Cav.) Trin. Ex Steud.^b^*Arundo donax* L.^a^
Bereztem	*Aristolochia paucinervis* Pomel	Aristolochiaceae	797A	11	Aorta palpitation	Common	*Aristolochia baetica* L ^a^*Aristolochia longa* L. ^a,c^
Fouilia	NA	NA	NA	11	Fractures	Common	---
Tasskra	*Echinops spinosissimus* subsp. *fontqueri* (Pau) Greuter	Asteraceae	478K	10	Rheumatism, Colds, Female reproductive ailments	Common	*Echinops spinosissimus* subsp. *fontqueri* (Pau) Greuter ^a,b,c^
Ouden helouf	*Pulicaria odora* (L.) Reichenb.	Asteraceae	806A	10	Gain weight, Sterility	Common	*Ranunculus bullatus* L.^a,c^
							*Ranunculus macrophyllus* Desf.^a,b^
							*Ranunculus muricatus* L.^a,c^
							*Ranunculus arvensis* L.^a^
Addad	*Carlina gummifera* (L.) Less.	Asteraceae	835A	10	Acne, Pruritus, Fumigation	Rare	*Atractylis gummifera* L. ^a,b,c^
Bekbouka	*Bunium bulbocastanum* L.	Apiaceae	92A	9	Gain weight	Rare	*Colchicum autumnale* L. ^a,b,c^
Amssekhsser	*Ammoides pusilla* (Brot.) Beistr.	Apiaceae	794A	9	Incense	Rare	*Polygonum aviculare* L.^a^
							*Polygonum equisetiforme* S.M.^a^
							*Polygonum maritimum* L.^a^
							*Daucus crinitus* Desf.^a^
Harmel	*Peganum harmala* L.	Zygophyllaceae	837A	9	Rheumatism	Common	*Peganum harmala* L*.*^a,b,c^
N’jem	*Cynodon dactylon* (L.) Pers.	Poacese	838A	8	Cold, Diuretic, Bladder infection	Common	*Cynodon dactylon* (L.) Pers. ^a,b,c^
Tafgha	*Rhaponticum acaule* L.	Asteraceae	842A	8	Stomach problems	Common	*Centaurea chamaerhaponticum* Bail.^a^
Tiguendizt	*Anacyclus pyrethrum* var. *pyrethrum Catannanche caerulea* L., *Anacyclus pyrethrum* var. *depressus* (Ball) Maire	Asteraceae	872A	7	Stomach problems, Chest pain, Rheumatism	Rare	*Anacyclus pyrethrum* (L.) Link ^a,b,c^
			805A				
			873A				
Ziyata	NA	NA	NA	6	Oral infection	Common	*Limoniastrum guyonianum* C.D.^a^
							*Limoniastrum ifniense* (Caball.) F.-Q. ^a^
							*Polygonum maritimum* L.^a, b^
Ghazghaz	*Carlina brachylepis* (Batt.) Meusel & Kästner	Asteraceae	791A	6	Coughing, Chest pain	Common	---
Deryass	*Thapsia villosa* L. *Thapsia transtagana* Brot.	Apiaceae	790A, 465 KGh	6	Aphrodisiac, Gain weight, Colds, Incense	Abundant	*Thapsia garganica* L.^a,b,c^
							*Thapsia villosa* L.^a^
Bllalourz	*Asphodelus* cf. *microcarpus* Parl.	Asphodelaceae	475K	6	Gain weight, Skin disease, Rheumatism, Colds	Abundant	*Asphodelus* cf. *microcarpus* Parl.^a,c^
							*Asphodelus ramosus* L.^a^
							*Asphodelus aestivus* Brot.^b^
							*Asphodelus cerasifer* J.Gay.^c^
Kelkh	*Ferula communis* L*.*	Apiaceae	840A	5	Sterility, Gain weight	Common	*Ferula communis* L*.*^*a,b,c*^
Bougoudz	*Tamus communis* L.	Dioscoraceae	796A	5	Skin diseases	Common	*---*
Khoudenjel	NA	NA	NA	5	Colds, Gain Weight	Common	*Alpinia officinarum* Hance ^*a,b,c*^
Taryala	NA	NA	NA	5	Aphrodisiac	Common	*Mandragora autumnalis* Bertol. ^*a,b,c*^
Tarra soudaniya	*Cyperus rotundus* L*.*	Cyperaceae	845A	4	Hair problems	Common	*Cyperus rotundus* L*.*^*a,b,c*^
							*Cyperus longus* L.^c^
							*Cyperus articulates* L.^a^
							*Cyperus maculates* Boeck.^a^
Tigheghcht	*Silene vulgaris* (Moench) Garcke	Caryophyllaceae	800A	4	Clean wool, Intestinal pains, Incense, Itching	Common	*Saponaria vaccaria* L.^a^
							*Saponaria glutinosa* Bieb.^a^
							*Silene inflata* Sm.^a^
D’bagh	*Quercus ilex* L. subsp. *rotundifolia* (Lam.) T. Morais	Fagaceae	486K	4	Stomach problems, Hair problems, Skin inflammation	Common	*Quercus ilex* L. subsp. *rotundifolia* (Lam.) T. Morais^a^
							*Quercus suber* L.^c^
Awedmi	*Armeria* cf. *alliacea* (Cav.) Hoffmanns. & Link, *Meum athamanticum* Jacq.	Plumbaginaceae	802A, 862A	4	Rheumatism	Rare	*Polygonum aviculare* L.^a^
							*Polygonum equisetiforme* S.M.^a^
							*Armeria* cf. *alliacea* (Cav.) Hoffmanns. & Link ^a^
							*Armeria mauritanica* Wallr.^a^
Boughlam sahraoui	*Spergularia marginata* (DC.) Kittel	Caryophyllaceae	869A	4	Cold, Gain weight	Rare	*Spergularia marginata* (DC.) Kittel ^a^
Telh	*Acacia gummifera* Willd.	Mimosaseae	846A	4	Fumigation	Common	*Acacia gummifera* Willd.^a^
							*Acacia raddiana* Savi.^c^
							*Acacia seyal* Del.^c^
M’ghizla	*Eryngium tricuspidatum* L.	Apiaceae	793A	3	Gain weight, Tonic, Rheumatism	Common	*Eryngium tricuspidatum* L.^a^
Swak Raayan	NA	NA	NA	3	Vitiligo	Common	---
Derdar	NA	NA	NA	3	Migraine	Common	---
Bessbess	*Foeniculum vulgare* P. Mill	Apiaceae	843A	3	Stomach problems, Sterility	Common	*Foeniculum vulgare* P. Mill ^a,b,c^
							*Foeniculum dulce* DC. ^a^
Hedja	NA	NA	NA	2	Rheumatism	Common	*Citrullus colocynthis* (L.) Schard. ^a,b,c^
Aarq sous	NA	NA	NA	2	Stomach and Throat problems	Common	*Glycyrrhiza glabra* L. ^a,b,c^ ,
							*Glycyrrhiza foetida* Desf. ^a,c^
Awermi (Fijel)	*Ruta montana* L.	Rutaceae	789A	2	Aphrodisiac, Rheumatism, Colds	Common	*Ruta montana* L. ^a,b,c^,
							*Ruta chalepensis* L. ^a,b,c^
							*Haplophyllum vermiculare* Hand. & Maz. ^a^
							*Haplophyllum tuberculatum* (Forssk.) A. Juss. ^b^
Boujlal	NA	NA	NA	2	Gain weight	Rare	---
Soussban	NA	NA	NA	2	Gain weight	Common	*Iris germanica* L. ^a,b,c^
							*Iris florentina* L. ^a^
							*Iris pseudoacorus* L. ^a^
Frifra	NA	NA	NA	2	NA	Rare	---
S’der	*Ziziphus lotus* (L.) Lam.	Rhamnaceae	489K	2	Stomach problems, Bladder problems	Common	*Ziziphus lotus* (L.) Lam. ^a,b,c^
Doum	*Chamaerops humilis* L.	Palmaceae	841A	2	Aphrodisiac	Abundant	*Chamaerops humilis* L.^a^
Bouzfour	*Ammoides pusilla* (Brot.) Beistr.	Apiaceae	474K	2	Incense	Rare	*Daucus crinitus* Desf. ^a^
Abu	*Kundmania sicula* (L.) DC.	Apiaceae	808A	2	Aphrodisiac, Sterility, Colds, Rheumatism	Common	*Thapsia garganica* L. ^a,b^
							*Thapsia villosa* L. ^a^
Kef saboun	NA	NA	NA	2	Colds	Rare	---
Kers aanou	NA	NA	NA	2	Aphrodisiac, Gain weight, Colds	Rare	---
Terta	NA	NA	NA	2	NA	Common	---
Belhdar	NA	NA	NA	2	NA	Rare	---
S’koum	*Asparagus stipularis* Forsk.	Asparagaceae	844A	2	Aphrodisiac	Common	*Asparagus stipularis* Forsk. ^a,b^
							*Asparagus albus* L. ^a^
							*Asparagus acutifollius* L. ^a^
							*Asparagus pastorianus* Webb. & Berth. ^a^
							*Asparagus altissimus* Munb. ^a^
R’tem	NA	NA	NA	1	Incense, Abortion	Rare	*Retama monosperma* Boiss. ^a^
							*Retama sphaerocarpa* (L.) Boiss. ^a^
							*Retama retam* (Forsk.) Webb. ^a^
Tizgha	NA	NA	NA	1	NA	Rare	---
Baguremane	NA	NA	NA	1	NA	Rare	---
Ghartague	NA	NA	NA	1	NA	Rare	---
Nedkhir	NA	NA	NA	1	NA	Rare	---
Temt	*Carthamus pinnatus* Desf.	Asteraceae	468K	1	Fumigation	Common	---
Swaka	*Juglan regia* L.	Juglandaceae	469K	1	Tooth care, Gingivitis	Abundant	*Juglan regia* L. ^a, b,c^
Smar	*Juncus maritimus* Lamk.	Juncaceae	839A	1	Cold, Fumigation	Common	*Juncus maritimus* Lamk.^a,b,c^
							*Juncus acutus* L. ^a,b^
							*Juncus bufonius* L. ^a,b^
Saleh n’der	*Verbascum sinuatum* L.	Scrofulariaceae	870A	1	Ophthalmopathy	Common	*Verbascum sinuatum* L.^a,b^
							*Verbascum thapsiforme* Scrad ^c^
							*Verbascum granatense* Boiss ^a^
Tizorin	*Valeriana tuberosa* L.	Valerianaceae	830A	0	Colic	Common	---
Azalim n’ouchen (Aansla)	*Drimia maritima* (L.) Stearn*,*	Hyacinthaceae	471K, 882 A	0	Hepatitis, Black magic, Rheumatism, Colds	Abundant	*Urginia maritime* (L.) Baker ^a,b, c^
							*Urginia noctiflora* Batt. & Trab. ^a^

NA: not locally available.

a. Bellakhdar [11].

b. Boulos [13].

c. Benchaabane et Abbad [12].

Of the 67 vernacular names recorded in the free lists, 57 were included for further analysis (Table 2) based on subsequent discussion with herbalists which revealed 10 remedies that were listed in a single free list but not confirmed by other herbalists (*defla, delya, jebouj, kalyptus, zitoun, klikha, louya, n’khal*) or that were common culinary species (*skenjbir* (*Zingiber officinale* Roscoe), *kherqoum beldi* (*Curcuma sp*)). Two additional vernacular names were identified though work with collectors (Table 2). We were able to collect and identify one or more species for 39 of the recorded vernacular names. The other 20 were not available for collection: reported by herbalists to be rare, extinct or imported.

We collected more than one species for 5 of the 39 vernacular names (*awedmi, azalim n’ouchen, deryass, serghina/tasserghint and tiguendizt*), one species collected under two different vernacular names (*Ammoides pusilla* (Brot.) Beistr. was collected as *amssekhsser* and *bouzfour*) and two varieties of *Anacyclus pyrethrum* were collected, resulting in a total of 43 species identified.

Of the underground plant parts collected for identification 92% were roots, 4% were rhizomes, 4% were bulbs. Asteraceae (9 species), Apiaceae (8 species) and Caryophyllaceae (4 species) plant families contained the highest number of species, with the remaining families containing 3 or fewer species each (Table 2).

### Comparison to the literature

We identified six new vernacular names (*belhdar, tizgha, baguremane, ghartague, nedkhir, tizorin*) which, to our knowledge, had not previously been reported in the literature as being used medicinally in Morocco (Table 2). Of the medicinal roots listed by vernacular names in Table 2, 17 (29%) had not been identified botanically in the literature. We identified 4 of these: *bougoudz* (*Tamus communis* L.), *ghazghaz* (*Carlina brachylepis* (Batt.) Meusel & Kästner), *tizorin* (*Valeriana tuberosa* L.) and *temt* (*Carthamus pinnatus* Desf.). Of the 42 vernacular names in Table 2 which had botanical identification listed in the most commonly cited literature, 25 of these had 2 or more species listed. Up to 5 species were reported for a single vernacular name (*s’koum*), with an average of 2.21 species listed per vernacular name. Of the 39 vernacular names we identified, at least one of the species we identified matched at least one of the names listed in the literature for 25 cases (64%); none of the species we collected matched any of the species listed in literature for 10 cases (26%); and there were no species listed in the literature for 4 cases (Table 2).

We were able to compare our 45 botanical identifications (43 species) to the species listed for their corresponding vernacular names in the literature for 41 cases (due to 4 not being previously identified). Our botanical identification matched at least one of the names listed in the literature 63% of the time (26 cases) and did not match any species listed in the literature 37% of the time (15 cases). There were 3 cases where the genus but not the species matched between the species we collected and those listed in the literature. Our botanical identification matched at least the genus of at least one of the names listed in the literature 71% of the time (29 cases); 29% of the time our identification did not match even the genus of any of the species listed in the literature (Table 2).

The three most commonly cited literature works we compared to were: Bellakhdar 1997 “La Pharmacopée Marociane Traditionelle: Médicine Arabe Ancienne et Savoirs Populaires” [11], Benchâabane and Abbad 1997 “Les plantes medicinales commercialisées à Marrakech” [12], and Boulos 1983 “Medicinal Plants of North Africa” [13]. Of our 45 botanical identification, 91% (41 cases) were listed by Bellakhdar 1997 and 64% (29 cases) each by Boulos 1983 and Benchâabane and Abbad 1997. Of the names provided by each, 63% (26 matches of the 41 listed) of the identifications provided by Bellakhdar 1997, 58.6% (17 matches of the 29 listed) of the identifications provided by Boulos 1983, and 55.2% (16 matches of the 29 listed) of those provided by Benchâabane and Abbad 1997 agreed with our identifications (Table 2). Considering these differing rates of agreement from a geographical perspective, we note that Bellakhdar 1997 covered all of Morocco, the broadest geographical region, Boulos 1983 worked in 13 of the provinces listed in Figure 1 (all of Morocco except the 3 most southerly provinces) and Benchâabane and Abbad 1997 worked only in Marrakech region (7 in Figure 1). Our study was conducted mainly in Marrakech and Azilal provinces (7 and 12 in Figure 1). There is thus geographical overlap between our study and each of the 3 most commonly used pieces of literature. Rather than seeing greater overlap in the work from a similarly focused geographic area (Benchâabane and Abbad 1997), we found the greatest overlap with the broader, more comprehensive work of Bellakhdar 1997. This may be due to the fact that Bellakhdar 1997 is a more comprehensive work, listing more species per vernacular name that other literature (Table 2). However there was still only 63% agreement between Bellakhdar 1997 and our botanical identifications, and 29% of the time our identification didn’t match even the genus of any of the species listed in the literature.

## Discussion

Botanical identification of remedies from roots, barks and resins remains a particularly challenging problem [7,39]. Several of the studies we reviewed that examined at the pharmacological and physiological function of species, had based the botanical identity of their species on identification from vernacular names using previous literature. This study has shown major inconsistencies in this method, rendering the above studies incomparable to any past or future studies.

Difficulty in identifying medicinal roots poses a problem not only to scientific research but also to local traditional medicine practitioners, who need to be able to identify roots to ensure effective and safe treatment of their clients. Poor identification of medicinal roots by both scientific research and local herbalists has a number of important implications.

There is increasing controversy surrounding substitution and adulteration of medicinal roots traded in the herbal markets of Morocco [40]. Poor identification of medicinal roots also has implications for toxicology and public health policy. Poisoning can occur due to misidentification by local practitioners, and the ability of scientists and public health officials to deal with poisoning cases is impaired when the scientific literature is inaccurate [40]. For instance, in the most commonly used literature, *bekbouka* is reported to be *Colchicum autumnale* L. (a highly toxics species that might lead to health complications if misused [4,41]), but we identified it as *Bunium bulbocatanum* L. Our molecular bar-coding work has identified extensive substitution of *Thapsia* spp. (reported as toxic by Bellakhdar) for multiple other roots [11,44]. A relevant database of vouchered medicinal roots should be a priority for scientists to ensure adequate botanical identification and a reliable source for public health officials and future research.

Many of the root species used medicinally in Morocco are collected from the wild creating the potential of overharvesting. Poor or inaccurate identification complicates conservation efforts for endangered and rare species. These species are sometimes traded under different vernacular names to obscure substitution and adulteration practices or to circumvent prohibitions on their collection and trade. For example, *tiguendizt* has been previously described mainly as *A. pyrethrum* but we revealed the existence of two varieties which are morphologically similar. Massive wild harvest targets *A. pyrethrum* var. *pyrethrum* and little information is available on the current state of wild populations. Proper identification will help ensure protection of these natural resources.

## Conclusion

Although DNA bar coding, which is emerging as a potential tool for plant identification, may prove useful to combat both problems of toxicity and overharvesting of endangered species [42], including medicinal plants used in Morocco [43,44], botanical identification remains a vital tool for research, conservation and public health and safety. In summary, a lack of voucher specimens of medicinal plants, particularly medicinal roots, means many studies have been dependent on the available literature which, we have shown, is not as complete as is needed. There is an urgent need for accurate botanical identification of wild medicinal plants. We identified the existence of cases where multiple species are categorized under the same vernacular name and we provide site-specific data on botanical identity of traditional medicinal plants. Differences in knowledge (e.g. between ethnic groups), habitat, and geographic distribution can alter the local nomenclature used for naming medicinal plants. This suggests a need for further exhaustive investigation targeting botanical identification (with voucher specimens) of medicinal plants collected and used across multiple regions of Morocco. Furthermore, special attention should be given to endangered and over harvested species to ensure their sustainable use.

## Competing interests

The authors declare that they have no competing interests.

## Authors’ contributions

All authors helped prepare the manuscript, and read and approved the final manuscript. Initial conceptualization of the larger project by GJM, AA, AO and others. AO and BP set the literature review key words and inclusion / exclusion criteria. The literature review was carried out by AO with help from GE. Data and specimen collection, and specimen identification by AO, Dr Mohamed Ibn Tattou and Dr Mohamed Fennane. Data synthesis by AO, with help from BP.
